# Knowledge discovery from high-frequency stream nitrate concentrations: hydrology and biology contributions

**DOI:** 10.1038/srep31536

**Published:** 2016-08-30

**Authors:** Alice H. Aubert, Michael C. Thrun, Lutz Breuer, Alfred Ultsch

**Affiliations:** 1 Institute for Landscape Ecology and Resources Management (ILR), Research Centre for BioSystems, Land Use and Nutrition (iFZ), Justus Liebig University Giessen, Heinrich-Buff-Ring 26, D-35392 Giessen, Germany; 2 Databionics, Mathematics and Computer Science, Philipps University Marburg, Hans-Meerwein-Strasse 6, D-35032 Marburg, Germany; 3 Centre for International Development and Environmental Research (ZEU), Justus Liebig University Giessen Goethestrasse 58, D-35390 Giessen, Germany.

## Abstract

High-frequency, *in-situ* monitoring provides large environmental datasets. These datasets will likely bring new insights in landscape functioning and process scale understanding. However, tailoring data analysis methods is necessary. Here, we detach our analysis from the usual temporal analysis performed in hydrology to determine if it is possible to infer general rules regarding hydrochemistry from available large datasets. We combined a 2-year in-stream nitrate concentration time series (time resolution of 15 min) with concurrent hydrological, meteorological and soil moisture data. We removed the low-frequency variations through low-pass filtering, which suppressed seasonality. We then analyzed the high-frequency variability component using Pareto Density Estimation, which to our knowledge has not been applied to hydrology. The resulting distribution of nitrate concentrations revealed three normally distributed modes: low, medium and high. Studying the environmental conditions for each mode revealed the main control of nitrate concentration: the saturation state of the riparian zone. We found low nitrate concentrations under conditions of hydrological connectivity and dominant denitrifying biological processes, and we found high nitrate concentrations under hydrological recession conditions and dominant nitrifying biological processes. These results generalize our understanding of hydro-biogeochemical nitrate flux controls and bring useful information to the development of nitrogen process-based models at the landscape scale.

Human activities modify the global nitrogen cycle, particularly through farming^1^. These practices have unintended consequences; for example, nitrate lost from terrestrial runoff to streams and estuaries can impact aquatic life^2^. Thus, studying nitrate export, i.e., nitrate concentrations at the outlet of a watershed, is a major concern. Existing labor-intensive monitoring strategies that have been in place for several decades have recently been complemented by the development of *in-situ* technologies that allow for high-frequency (sub-hourly) sampling. High-frequency monitoring has been shown to be a beneficial addition to the previous lower frequency monitoring schemes^3^. A decade ago, high-frequency monitoring was expected to bring new insights into watersheds functioning^4^, and indeed, it has helped^5^ identify sources and transport pathways of nutrients^6^ and quantify processes and metabolisms of coupled nutrients^7^ across multiple time scales^8^. This has allowed researchers to disentangle the effects of multiple processes^9^. Now, the availability of several year-long high-frequency datasets invites the application of data mining techniques^10^.

Catchments are dynamic systems, and present observations rely on previous hydrological states. In water sciences, data are mostly analyzed with respect to time. Analyses focus on either long-term, seasonal, or short-term variations, including fluctuations resulting from flood events or diurnal cycles. Temporal data structure is regularly analyzed in the time and frequency domain by time series decomposition^11^,^12^ and spectral analysis^8^,^13^,^14^,^15^, respectively. These methods are used to identify cycles and variability in the main transfer processes. Time-variant process modelling also allows us to explain the old water paradox^16^,^17^,^18^.

In this study, we look at nitrate concentration data differently by neglecting its temporal component in the data analysis. This approach is now possible and worth considering given the availability of datasets large enough to mine and expand our general knowledge. Data structures have already been studied independently of time, e.g., plotting a variable of interest against another variable. For example, correlating observed nitrate concentration with a simulated index based on the watershed wetness state^19^ refined the flushing hypothesis, and relating production of nitrogen gaseous species to the percent of the soil’s pore volume filled by water^20^ defined a conceptual model of nitrogen oxide emissions from soil. These non-temporal data structure analyses have brought new insights in watershed functioning and the nitrogen cycle.

Non-temporal data structure analysis was also used to compare high- and low-frequency monitoring data^3^. There, probability density functions (PDF, a function describing the likelihood a variable can take a given value) were estimated using kernel density estimators. However, as in any PDF estimation method, one of the critical parameters, the kernel width, was left undetermined. If too large of a kernel width is chosen, important structures may be undetected. Likewise, if the kernel width is too small, random fluctuations are overemphasized. To avoid an unclear choice in kernel width, we used the Pareto Density Estimation (PDE), in which kernel width has proved to be particularly suitable for detecting modes in continuous data. PDE is particularly suitable for the discovery of mixtures of Gaussians^21^, but in the case of skewed distributions, transforming the data is required beforehand. In other scientific domains, thorough analysis of data structure focused primarily on the estimation of the PDF using the Pareto Density Estimation (PDE) of a variable of interest^21^,^22^.

The objective of this study is to generalize–or cast doubt on–the current understanding of nitrate fluxes at the catchment scale. At this point, in-stream nitrate concentrations in an agricultural catchment are mainly described in relation to time. Seasonal and event-related variations in nitrogen sources and transport processes throughout the year or during a wetting-drying cycle confer seasonal and short-term fluctuations to nitrate concentrations. To avoid the tendency of reinforcing the understanding of already described relationships, we included all measured variables from the catchment^23^. This naïve look at the data is common in data mining. We focused on the shape of the PDFs from high-frequency nitrate concentrations monitored in a 3.7-km^2^ mixed-land use catchment. Environmental variables (discharge, groundwater depth, soil moisture, soil temperature, stream temperature, stream conductivity, rainfall, air temperature and solar radiation) were considered as potential explanatory variables and were used in the process of knowledge discovery to identify the drivers of nitrate fluctuations in the catchment. Particularly, we were interested in whether these drivers are the same for low and high nitrate concentrations, as this result may assist in refining mechanistic models of nitrate fluxes.

## Results

### The large dataset

**Figure 1:**
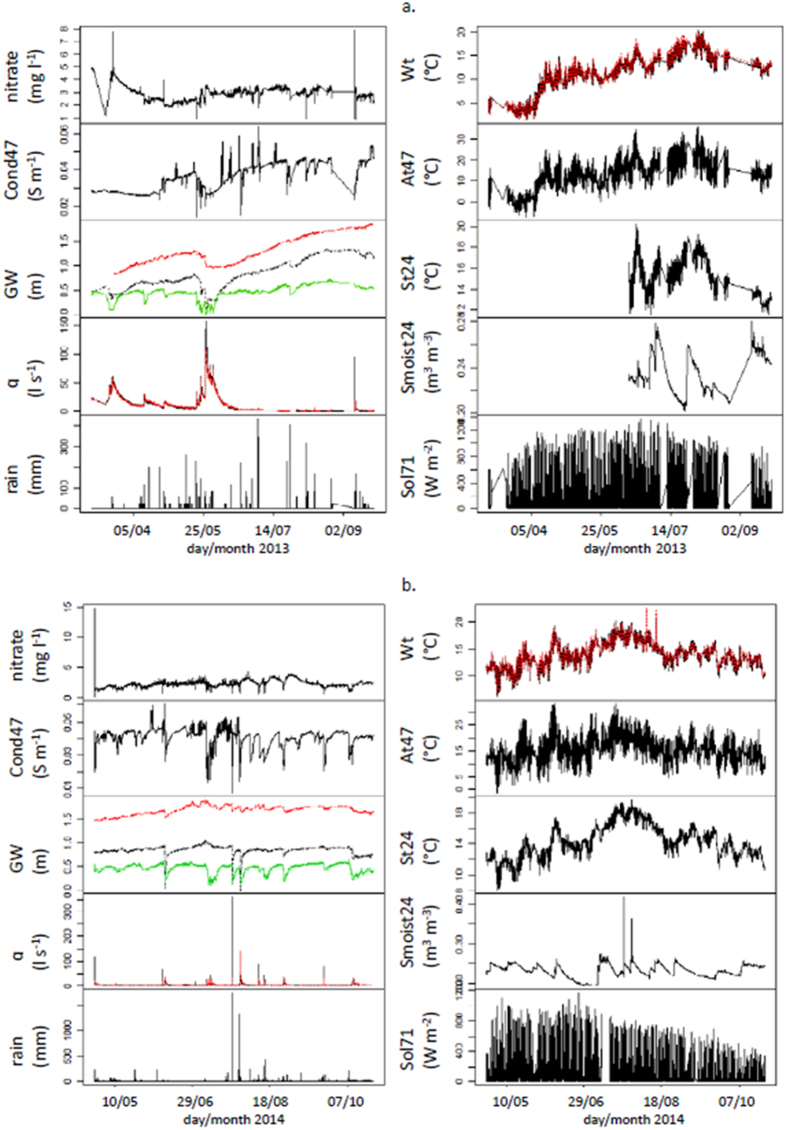
Times series of data, including nitrate, groundwater depth (GW) (lowland GW3 (black dotted curve), hillslope GW25 (red dashed), and riparian zone GW32 (green solid)), discharges (at the outlet q13 (black solid curve) and up-stream q18 (red dashed)), water temperature (Wt) (at the outlet (black solidcurve) and up-stream (red dashed)), soil temperature (St24), air temperature (At47), soil moisture (Smoist24), solar radiation (Sol71) and precipitation (rain) for 2013 (**a**) and 2014 (**b**).

The available dataset contained in total 32,196 data points for each of the 14 variables (in total, 4% missing data), making it suitable for data mining (pp. 243 of 24). The raw time series for each year are presented in Fig. 1. For technical reasons, no nitrate data were available during winter, so the actual time span of nitrate monitoring was 05 March 2013 12:45 to 24 September 2013 12:30 and 27 April 2014 00:00 to 23 October 13:15. Data were analyzed as a whole, without differentiating between the hydrological years. To do this, we filtered out the seasonal variation (see methods). Hereafter, when referring to sub-daily high-frequency fluctuations (after seasonal variation removal) a tilde (~) superscript will be added to the variable names.

### High-frequency nitrate data: a composite of the three modes

**Figure 2:**
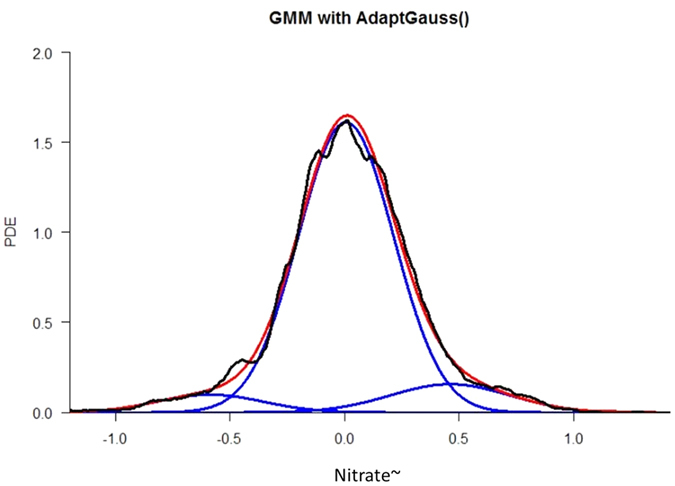
Component Gaussian Mixture Model (GMM) (blue lines), superposition of the Gaussians (GMM) (red line), and the PDF describing sub-daily nitrate~ in-stream concentrations (black line). The three modes (blue lines) of the GMM describe low (left, mean = −0.59, SD = 0.23), typical (centered, mean = 0.0053, SD = 0.21) and high (right, mean = 0.46, SD = 0.26) nitrate~ concentrations. Prior to data analysis nitrate concentrations were split by applying a two-component model, which describes seasonal and sub-daily fluctuations.

The PDF of the empirical values of nitrate~ concentration from the Vollnkirchener Bach watershed was modelled using PDE, resulting in three distinctive modes (see methods). The estimation of the empirical distribution (black curve) was modelled (red curve) using a Gaussian mixture model (GMM) composed of three Gaussians (blue curves) (Fig. 2). The goodness of fit was visualized with a quantile-quantile plot (Supplementary Fig. S1) and verified statistically with the Xi-Quadrat test (p = 1e-05) and Kolmogorov–Smirnov test (p < 1e-10). The central Gaussian represented typical nitrate~ concentrations, while the left and right Gaussians described the lower and higher concentrations of nitrate~, respectively. Bayes Theorem was used to calculate the class posterior probabilities. This calculation classified nitrate~ into the three classes: low (5% of the data), typical (89% of the data), and high nitrate~ concentrations (6% of the data).

### Modes characterized by environmental conditions

**Figure 3:**
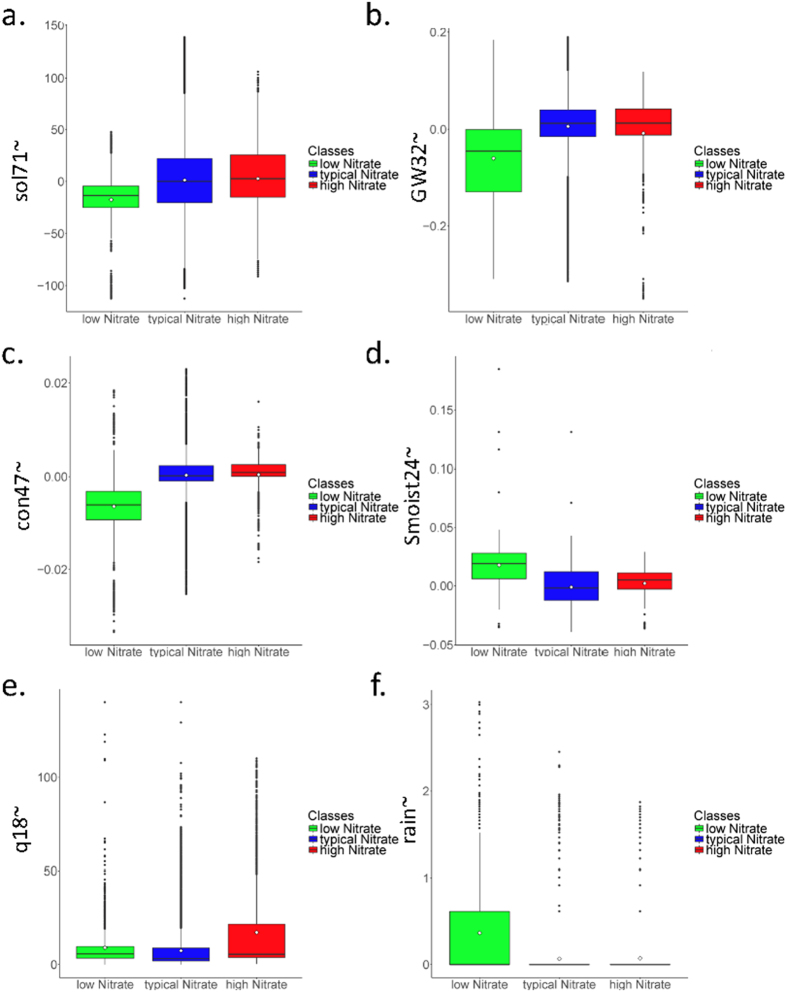
Boxplots of each nitrate~ concentration mode for the following environmental variables (**a**) solar radiation (Sol71~), (**b**) groundwater head in the riparian zone (GW32~), (**c**) conductivity (Con47~), (**d**) soil moisture (Smoist24~), (**e**) discharge (q18~) and (**f**) rainfall intensity (rain~). The white dot shows the arithmetic mean that was statistically tested using the t-test (Table 1). Original PDE curves for all variables are presented in the Supplementary Information (Fig. S2).

We compared the concurrently measured environmental variables for each mode of nitrate~. Lower nitrate~ was characterized by more superficial groundwater depth (GW32~), higher soil moisture (Smoist24~) and, on average, lower solar radiation (Sol71~) (Fig. 3, Table 1). High nitrate~ was characterized by deeper groundwater depth (GW32~), moderate soil moisture (Smoist24~) and, on average, higher solar radiation (Sol71~) than the low nitrate~ Gaussian curves. The typical nitrate~ class was quite similar to the high nitrate~ class, but the high nitrate~ was associated with more humid soils.

## Discussion

### Variables driving rapid nitrate fluctuations

The three depths of groundwater (GW3~, GW25~ and GW32~) represent the typical range in the spatial variability of the groundwater table conditions. They are located in the mid-reach lowland meadow (GW3), the cultivated land on the hillslope (GW25), and in a riparian meadow where a temporary tributary joins the stream (GW32)^25^. The riparian meadow groundwater depth was always selected as a driver of nitrate~. This result supports the importance of near-stream zones that are often reported as having a major impact on stream water quality^26^,^27^. GW32~ shows little seasonal fluctuation; most of the time, this groundwater depth remains high, which reflects connectivity to the stream network. This location is also more reactive than the other piezometers (Fig. 1). Conversely, the hillslope and lowland meadow groundwater depths are less reactive and fluctuate seasonally with a high amplitude. These locations have little influence on nitrate~ concentrations. The short-term nitrate fluctuations support the assumption that the constantly connected landscape elements are a major determinant of the high-frequency variability of solutes. Landscape elements for which connectivity exhibits low frequency fluctuation of a high amplitude are not predominant for stream water chemistry at the fine time scale^28^,^29^. Soil moisture (Smoist24~) was determined to be another major driver of nitrate~ concentrations in the high nitrate mode, supporting the results of previous studies^30^,^31^. Discharge also impacted nitrate~. Last, electric conductivity (cond47~) generally follows nitrate~ concentrations: when nitrate~ is low, conductivity (which also accounts for nitrate salts) is low.

Generally, air-, soil- and stream- temperatures~ are not meaningful to explain the high frequency fluctuations in water chemistry. Air, soil and stream temperatures showed primarily low-frequency fluctuations, aligning with variables such as groundwater depth in the agricultural hillslope, and the temperature data formed almost perfect Gaussians. Perfect Gaussians characterize variables with a clear combination of sinusoidal patterns, for both seasonal and diurnal time scales. Rainfall intensity (rain~) was not meaningful to explain the high frequency fluctuations in nitrate~ either. The lack of relation between rainfall and nitrate~ supports the findings of a previous study^32^ performed in the catchment using isotopic signatures. In the isotopic study, stream water was found to be more similar to groundwater than to rain water or soil water, illustrating the “old water paradox”^16^,^33^ once more, where old water flows during storm events. Rain and high celerity (the speed at which the perturbation wave is transmitted) lead to a reactive stream water level; however, it does not imply that rainwater is transported directly to the stream, where it could affect nitrate. Stream flow velocity, defining chemical transport, is, by definition, lower than celerity^34^.

### Combined effect of hydrology and biology on nitrate~

The low nitrate~ mode (left curve, Fig. 2) is driven by groundwater depth close to the surface and high soil moisture (Fig. 3), indicating the subsurface is saturated and hydrologically connected. Moreover, under such wet conditions, denitrification might be the most active biological process, adding to the importance of the hydrological state. We conclude that the low nitrate~ mode is defined by hydrological connectivity and a dominance of the denitrifying biological activity, that is, by a saturated catchment.

The high nitrate~ mode (right curve, Fig. 2) is driven by high solar radiation and deep groundwater, but soils are still moist. High solar radiation could suggest high evapotranspiration, given that moist soil indicates water is sufficiently available to plants. This behavior is typical for drying conditions. The drying phase has been linked to biological changes in the microbial community of soil aggregates^35^. Microbial diversity should increase with drying. When nutrient transport is reduced because of limited diffusion and the gaseous phase in the pores becomes important, anaerobic and aerobic communities will likely coexist. Thus, the denitrifying community is no longer the only one active; nitrification can occur. This balance between nitrification and denitrification could lead to the production and build-up of nitrate in the soil. This nitrate can then be mobilized during low intensity rain events. We interpret that high levels of nitrate~ are defined by hydrological recession and biologically active soils, where nitrification dominates.

The tipping-point, threshold or “hot moment”^26^, when biological drivers over-take hydrological drivers, is still unclear and needs to be determined in future work. The shift from denitrification to nitrification dominance also needs further data-based research. These interpretations align with models based on hydrological storage, distinguishing celerity and velocity^17^,^36^,^37^,^38^; however, these hydrological models were developed for conservative tracers, such as chloride. Our work highlights the need to add biological processes to hydrological models to allow for the production and consumption of chemicals, such as nitrate, known to strongly affect our environment in some regions.

### A method for initial data exploration

Our goal is to draw attention to the benefits of thorough analyses of large environmental datasets. In this case study, we show that new knowledge can be mined from empirical PDEs, thus we recommend data mining as a first step to understanding the driving forces in a catchment because it can provide a simplified, non-temporal view of solute export. This approach provides a glimpse of the catchment’s behavior, which is the compilation of many processes, by making use of low-flow data as well as storm-flow data^27^. By considering the variable of interest, in this case nitrate~, as non-temporal, the system was simplified and data structuration was observed^21^.

Data mining revealed a differentiation in nitrate~ modes and differences in underlying conditions. The roles of hydrology and soil microbiology in controlling nitrate~ were highlighted. Low nitrate~ occurred under hydrological connectivity and microbial denitrification. High nitrate~ occurred under hydrological recession and nitrifying conditions. The highly fluctuating component of the nitrate concentrations seems to be influenced by the saturation state of the catchment, although the seasonal component, which is known to be driven by saturation state, was removed. We are confident that other datasets analyzed with the described methods herein will produce strong advances in the interpretation of catchment hydro-biogeochemical processes. In future high frequency monitoring work, it will be important to monitor variables that allow the identification of the various biological processes occurring in the soil and in-stream, as the latter are reported to dampen terrestrial signals^39^. The difficulty will be to find variables that can be easily and cost-effectively measured at high temporal resolution. Potential biological variables include in-stream measurements of biological oxygen demand (BOD) and soil redox potential. We showed that connectivity plays an important role in nitrate concentrations; therefore, the identification of contributing (or connected) areas as well as the spatial identification of controlling variables would shed further light on solute export^40^. In the future, the development of networks of sensors^41^ or the use of high-temporal sensing distributed throughout a catchment^42^ could help to overcome these limitations.

## Methods

Nitrate concentration data were collected for two years in the Vollnkirchener Bach watershed, which is nested in the Critical Zone Observatory of the Schwingbachtal, in central Germany (references and data available at http://fb09-pasig.umwelt.uni-giessen.de:8081/). Technical issues and data checking reduced the time span to two growing seasons (05 March 2013 to 24 September 2013, n = 15,475 measurements and 27 April 2014 to 23 October 2014 n = 16,721 measurements, in total n = 32,196 measurements). Land-use is dominated by agricultural land and forests, covering 44 and 48% of the catchment, respectively. An *in-situ* hyperspectral UV-spectrometer (ProPS, Trios, Rastede, Germany, wavelength range 200–360 nm, path length 5 mm, solar panel supplied) measured absorption spectra every 15 min after a 5 s air blast to prevent the optics from biofouling. Wavelengths of 200–220 nm allowed the calculation of nitrate concentration, using a calibration adapted to the stream water’s baseline chemical composition. More detailed information on the calibration and the nitrate data checking is reported in ref. 43.

Other variables were monitored at high-frequency and used to explain the variations in nitrate, as they depicted the catchment state. Discharge (q, l s^−1^) and water temperature (Wt, °C) were measured at two gauging stations q13/Wt13 at the outlet and q18/Wt18 upstream and were measured every 5 min by pressure transducers (Diver DCX, Schlumberger Water Services, ON, Canada). Groundwater depth (GW, m) at three wells (GW25 on the hillslope, GW3 in lowland and GW32 in the riparian zone) were measured every 10 min by pressure transducers. Meteorology, i.e., air temperature (At47, °C), solar radiation (Sol71, W m^2^) and rainfall intensity (rain, mm), was captured every 5 min at a climate station 4 km from the outlet (Campbell Scientific Inc., CR1000 data logger, Loughborough, UK). Soil moisture (Smoist24, m^3^ m^−3^) and soil temperature (St24, °C) were measured hourly at 0.1 m depth, in the riparian zone, by electromagnetic induction (5TE sensors, EM50 data logger, Decagon, Labcell LTD, Alton, UK) beginning on 14 June 2013. Some of these variables are expected to directly influence nitrate in-stream concentrations, such as groundwater depths or rainfall intensity. Others are considered as proxies for biological activity, such as temperature and soil moisture, and evapotranspiration, the variable solar radiation^43^.

All time series were detrended to create a joint data set for both years. This process allowed the analysis of rapid fluctuations in the variables and considered both growing seasons at once. A two-component model with the variable-baseline subtracted from the raw time series was applied to obtain the high-frequency component of the variables. The variable-baseline was calculated using a low pass filter as a Fourier Transformation; the filter was set to 50 days. Thus, the high-frequency component is composed of fluctuations below the monthly time scale, down to 15 min. This residual time series is interpreted as a rapid and high-temporal fluctuation and is marked with a tilde (~) throughout the manuscript. Discharges and rainfall, which were typical of a reactive catchment, presented a seasonal baseline set to zero.

We then focused on our variable of interest: nitrate. First, we modelled the nitrate with three distinctive modes using the Adapt Gauss toolbox^22^ as shown in Fig. 2. The Adapt Gauss^17^ toolbox in R package allows for the modelling and verification of possible multimodal distributions as a mixture of Gaussian components. This approach is called Gaussian Mixture Modelling (GMM). Verification of the model was performed visually using a QQplot (Fig. S1A) and statistically with a Xi-Quadrat test (p < 1e-05) and a Kolmogorov–Smirnov test (p < 1e-10). In other words, GMM was constructed to fit the nitrate’s empirical PDF. The number of modes was calculated as the minimum of the Akaike Information Criterion (AIC) and the Bayesian Information Criterion (BIC)^44^. AIC and BIC were computed for the GMM with one to ten modes using an Expectation-Maximization (EM) optimization in the R package mclust^45^ (Fig. S1B). AIC and BIC were both in agreement. EM fitting, using a user-defined starting point in the Adapt Gauss toolbox^22^, resulted in better values for AIC and BIC. The GMM was supported by goodness-of-fit checks. These checks resulted in three different Gaussians for high-frequency nitrate~ (Fig. S1B). Bayes Theorem was used to calculate the class posterior probabilities.

The data were then mined to address if there were different drivers for the high and low nitrate~ concentrations. All data points for the potentially related variables were grouped according to the synchronous nitrate mode into the same three distributions: low, typical and high with respect to nitrate. We compared the three distributions for each variable visually using the PDE^21^ (Supplementary Information, Fig. S2), using boxplots resulting from the PDE (Fig. 3), and statistically using a Bonferroni corrected two-sample t-test for unequal sample sizes and unequal variances (Table 1). We only considered the variables that showed visually and statistically significant (p-values > 0.01) differences between modes in our interpretation.

## Additional Information

**How to cite this article**: Aubert, A. H. *et al.* Knowledge discovery from high-frequency stream nitrate concentrations: hydrology and biology contributions. *Sci. Rep.* **6**, 31536; doi: 10.1038/srep31536 (2016).

## Supplementary Material

Supplementary Information

## Figures and Tables

**Table 1:** The p-values of the differences between environmental conditions corresponding to each nitrate~ mode (low-typical, low-high, typical-high) are calculated by the Bonferroni corrected two-sample t-test with unequal variances, where “n.s.” indicates a non-significant result.

Variable	Low-typical	Low-high	Typical-high
GW3~	n.s.	5.7e-10	2.9e-10
GW32~	1.9e-137	2.8e-57	n.s.
Wt13~	n.s.	3.4e-09	n.s.
Sol71~	5.1e-111	2.5e-81	n.s.
Con47~	1.3e-205	6e-198	n.s.
Smoist24~	3.5e-220	1.2e-125	1.5e-15
q13~	n.s.	2.3e-31	3.2e-59
q18~	n.s.	9.6e-40	6.9e-69
rain~	n.s.	n.s.	n.s.

Only the variables with significant p-values and visual agreement using the class-wise Pareto Density Estimation are presented. Groundwater depth on the hillslope (GW25~) and water temperature at the upper gauge (Wt18~) are thus not presented.
