# Supplementary material for: Knowledge discovery from high-frequency stream nitrate concentrations: hydrology and biology contributions
Source: Sci Rep. 2016 Aug 30;6:31536. doi: 10.1038/srep31536 (PMC5004126; doi:10.1038/srep31536)

Scientific reports, category Earth and environmental sciences

Supplementary Information

Knowledge discovery from high-frequency stream nitrate concentrations: hydrology and biology contributions

Alice H. AUBERT[1,2*], Michael C. THRUN[3], Lutz BREUER[1,4], Alfred ULTSCH[3]

[1] Institute for Landscape Ecology and Resources Management (ILR), Research Centre for BioSystems, Land Use and Nutrition (IFZ), Justus Liebig University Giessen, Heinrich-Buff-Ring 26, D-35392 Giessen, Germany

[2] Now at: Eawag - Swiss Federal Institute of Aquatic Science and Technology

[3] Databionics, Mathematics and Computer Science, Philips University Marburg, Hans-Meerwein-Strasse 6, D-35032 Marburg, Germany

[4] Centre for International Development and Environmental Research, Justus Liebig University Gießen

*corresponding author's e-mail address: alice.aubert@eawag.ch

Subject areas

Water sciences, biogeochemistry, and applied mathematics

Supplementary Figure S1. A) Quantile-quantile plot for visual verification of the fit between modified PDE and the empirical nitrate-Extra distribution (Fig 2 of the main paper)

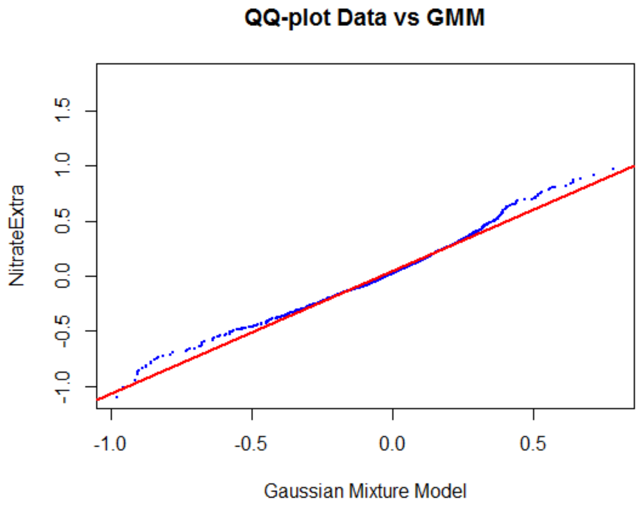

Supplementary Figure S1. B) Akaike information criterion of the GMM compared with EM computed models (R package mcclust) with 1 to 10 modes. Models with 3 Gaussians are preferable and the above shown GMM in A) is even better. (Fig 4 of the main paper)

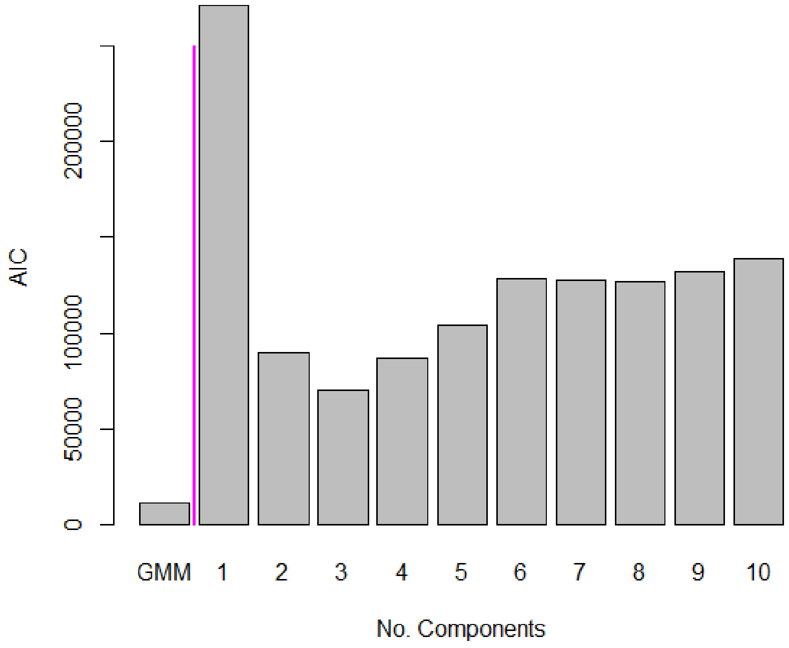

Supplementary Figure S2. Pareto Density Estimation for each mode of nitrate-Extra for the environmental variables

a. Groundwater level belowground in the lowland (GWl3)

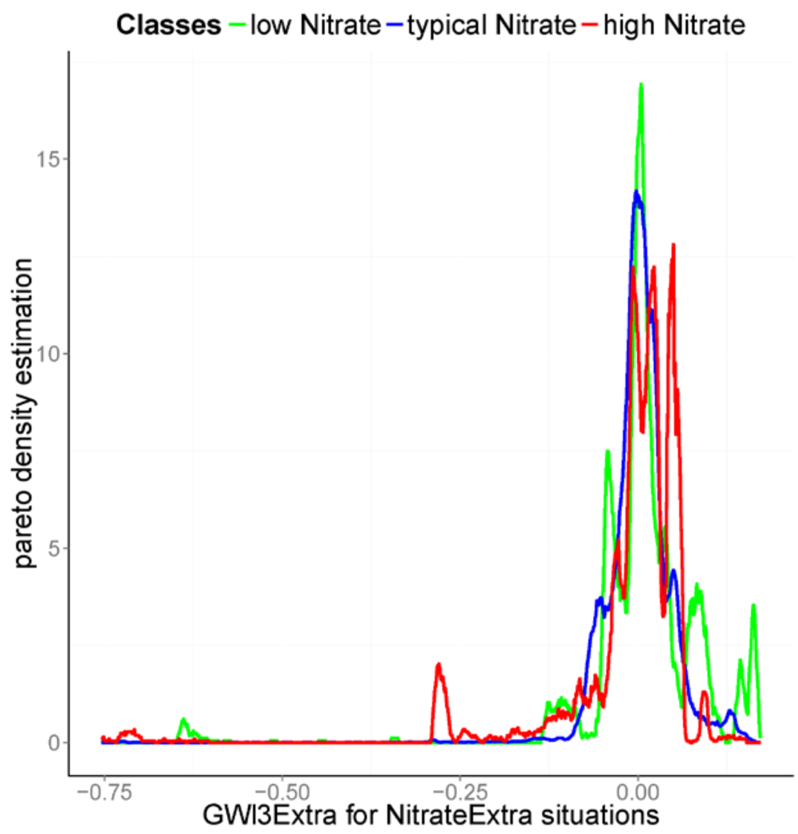

b. Groundwater level belowground on the hillslope (GWl25)

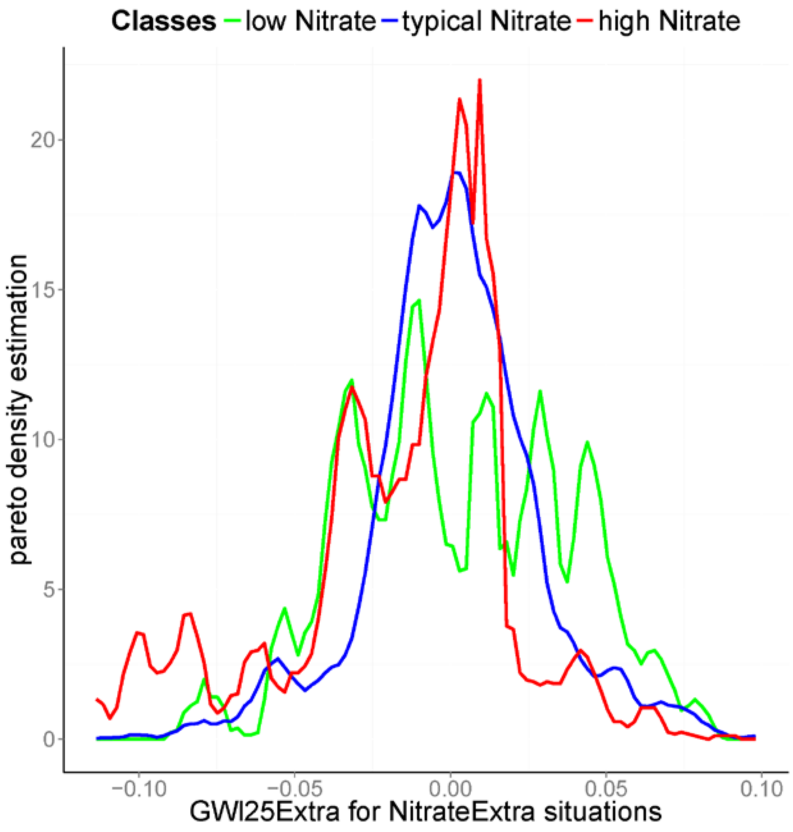

c. Groundwater level belowground in the lowland (GWl3)

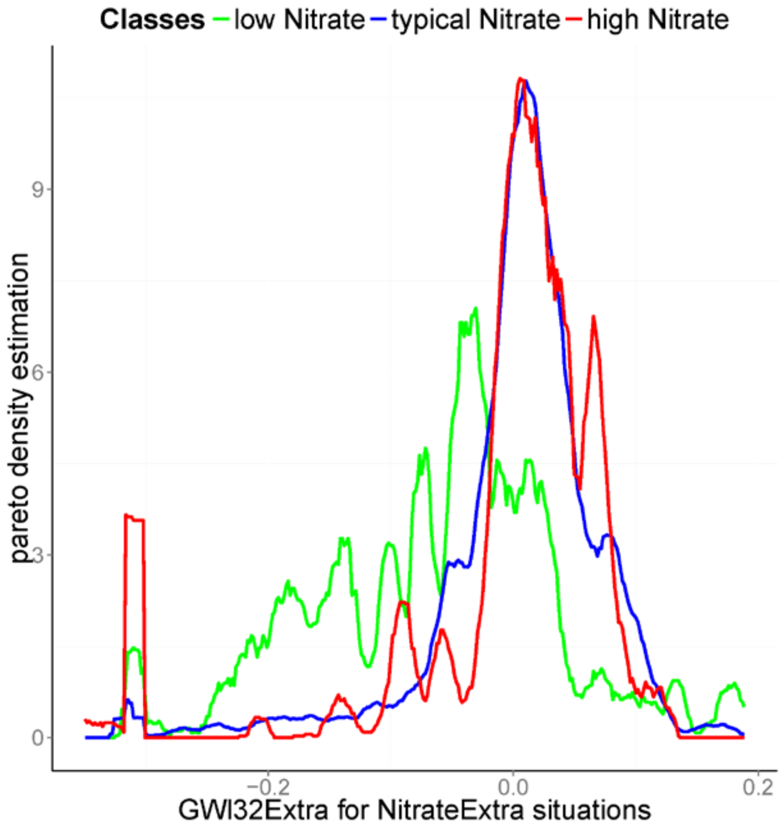

d. Water (stream) temperature at the outlet

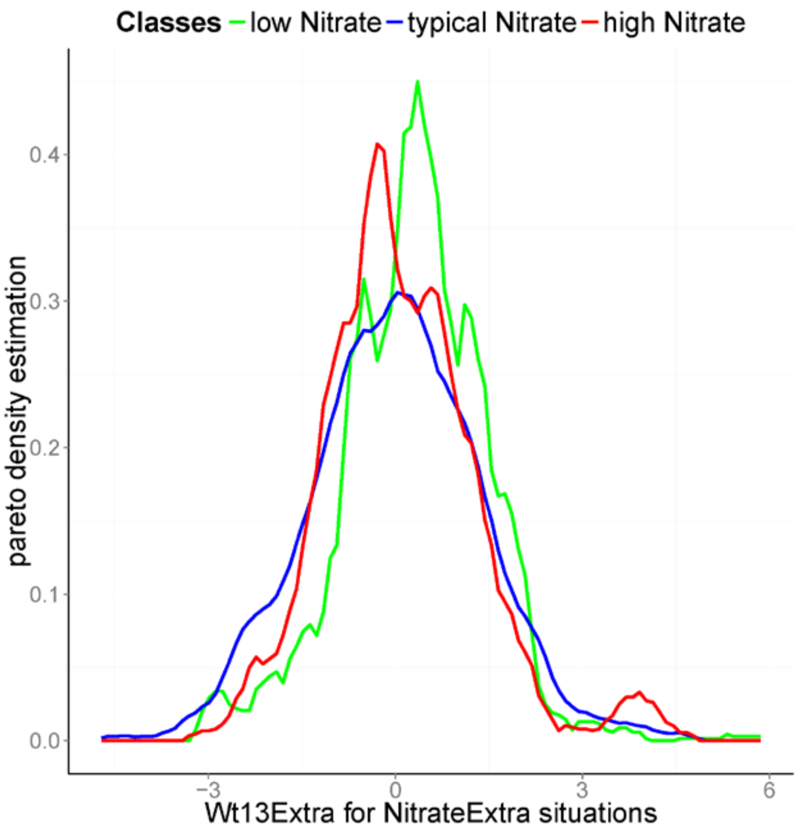

e. Water (stream) temperature upstream

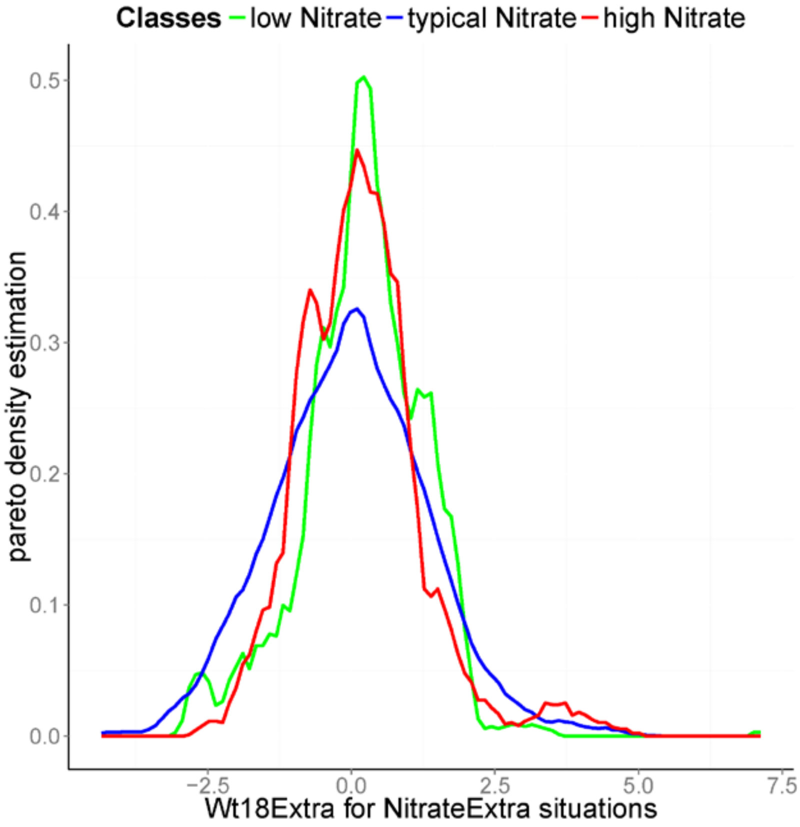

f. Solar radiation

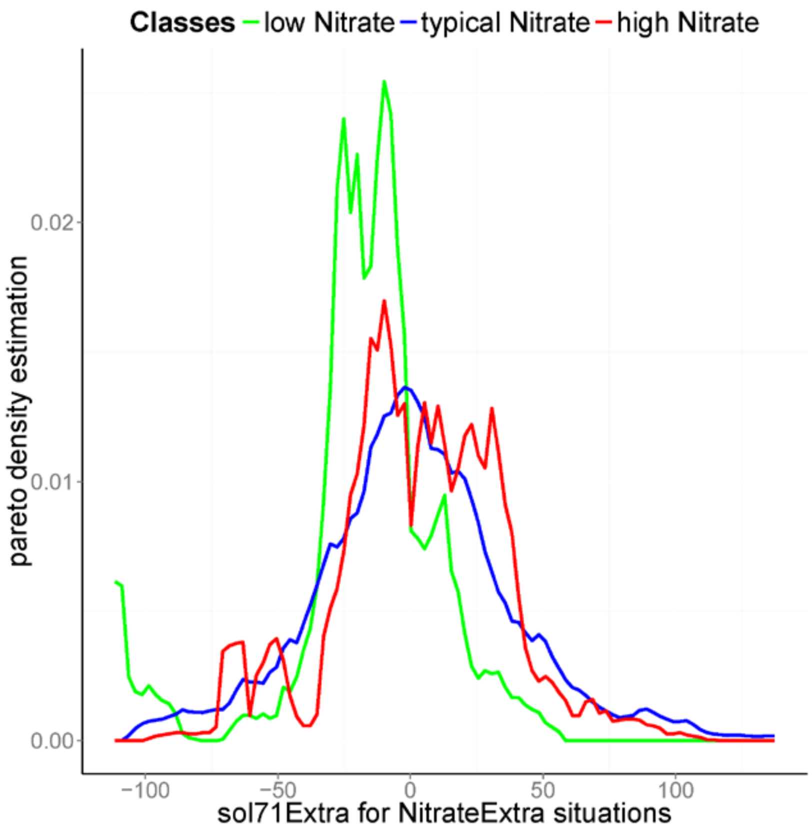

g. Conductivity in the stream

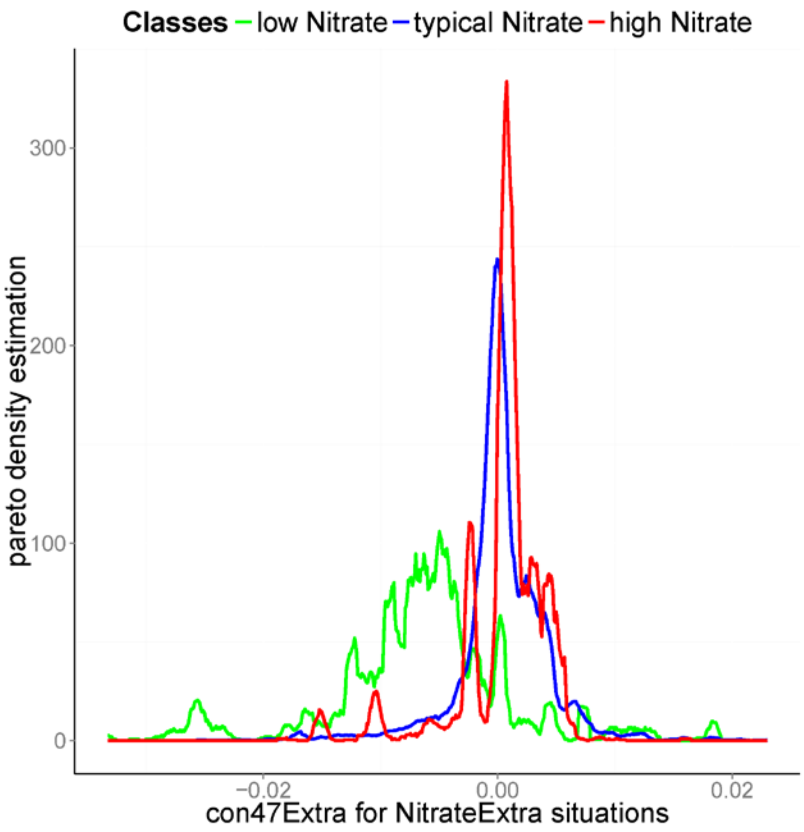

h. Air temperature

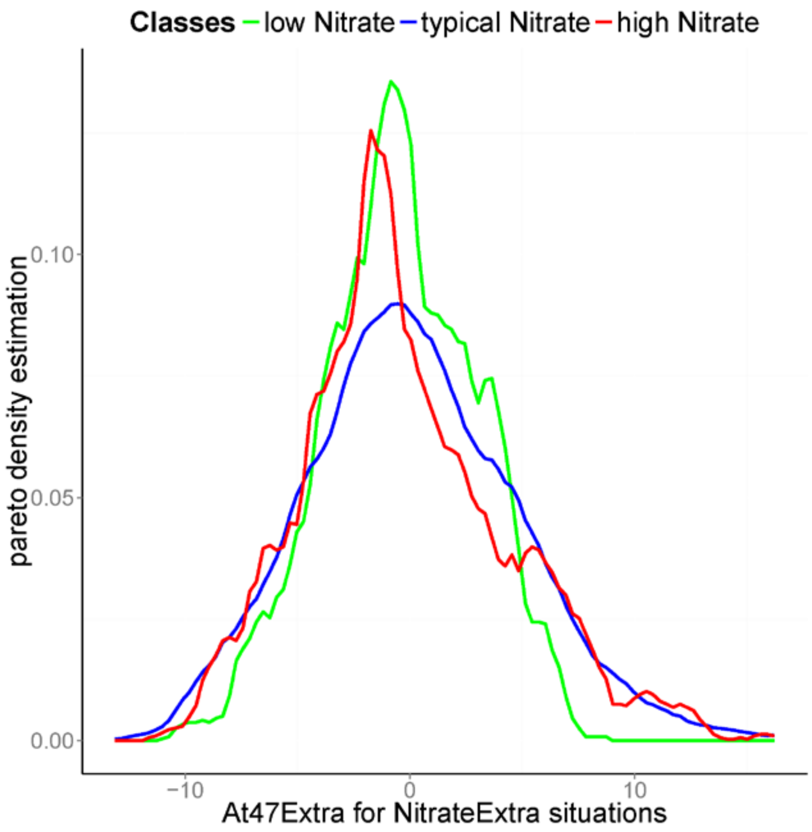

i. Soil moisture

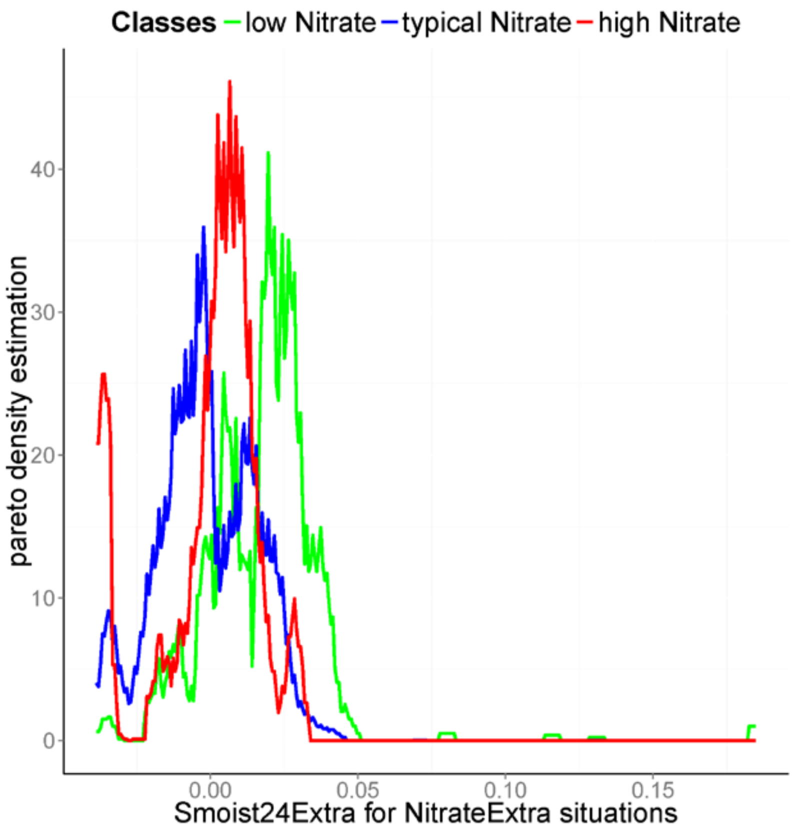

j. Soil temperature

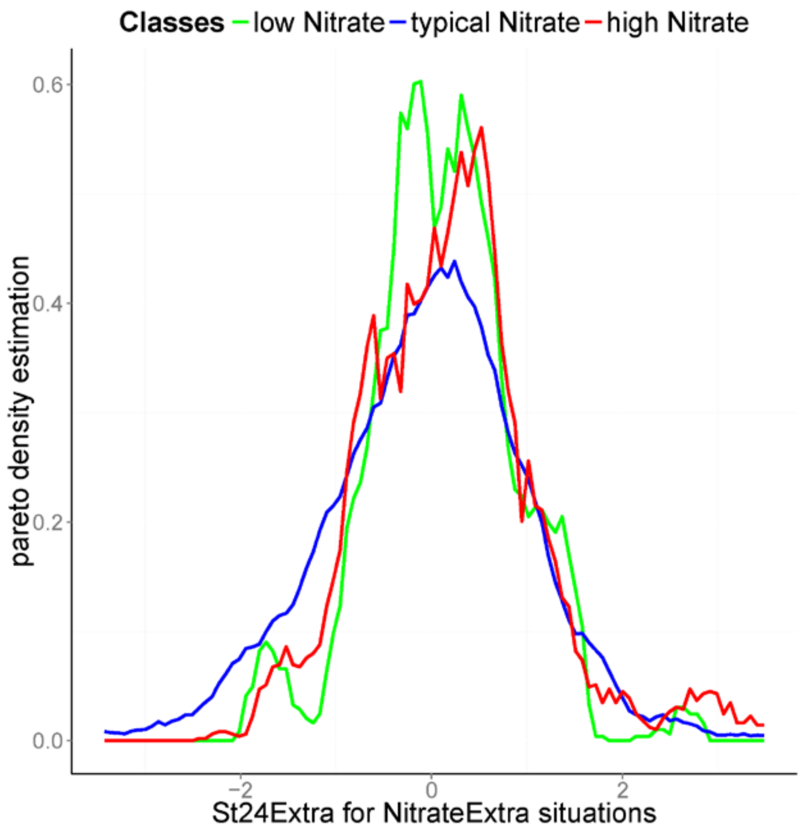

k. Discharge at the outlet

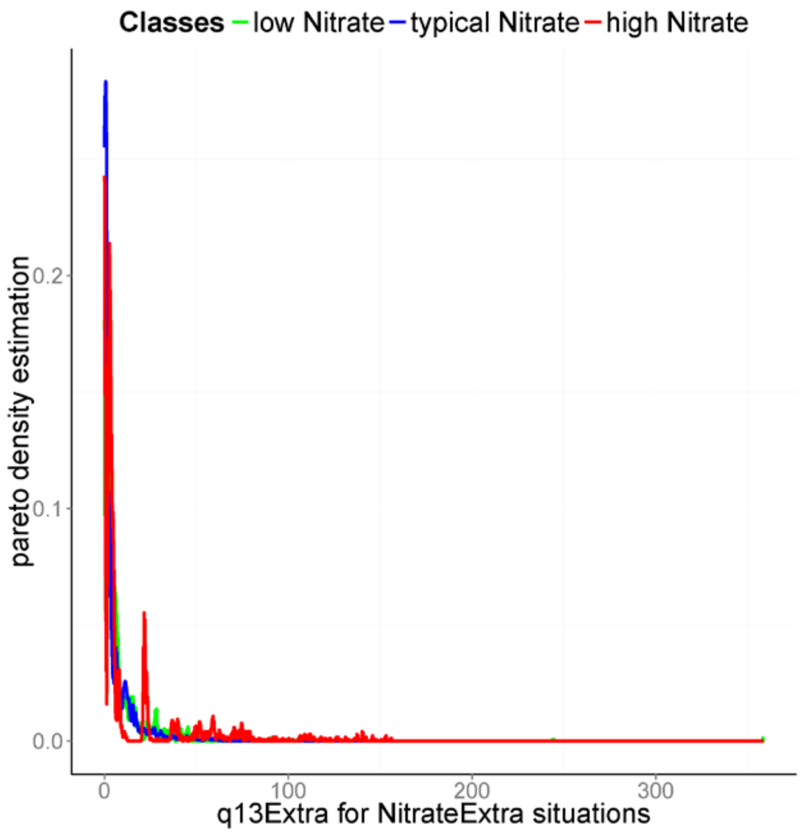

l. Discharge upstream

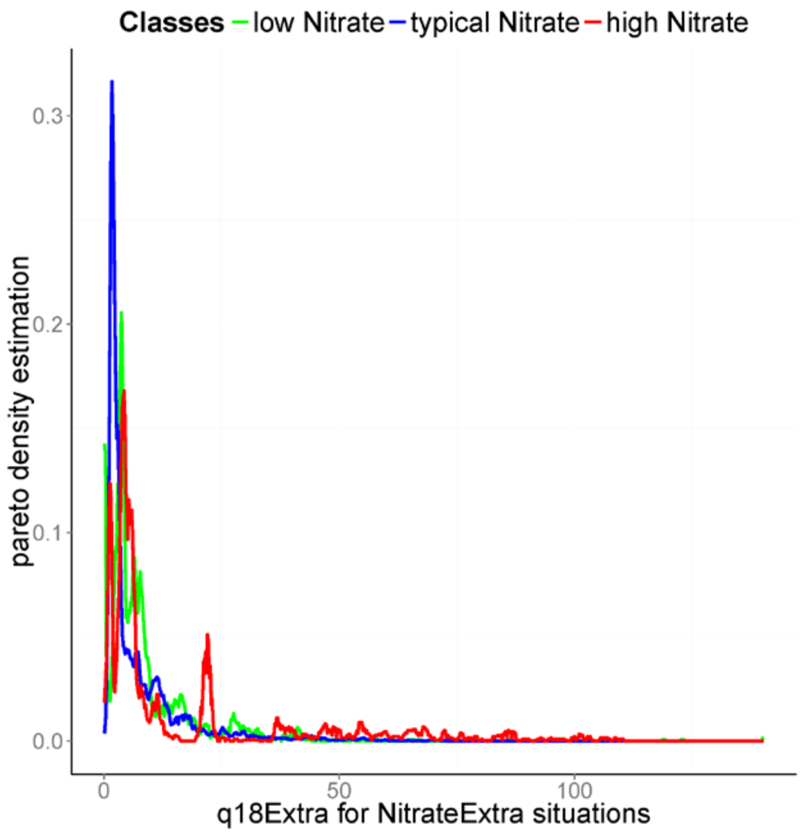

m. Rainfall intensity

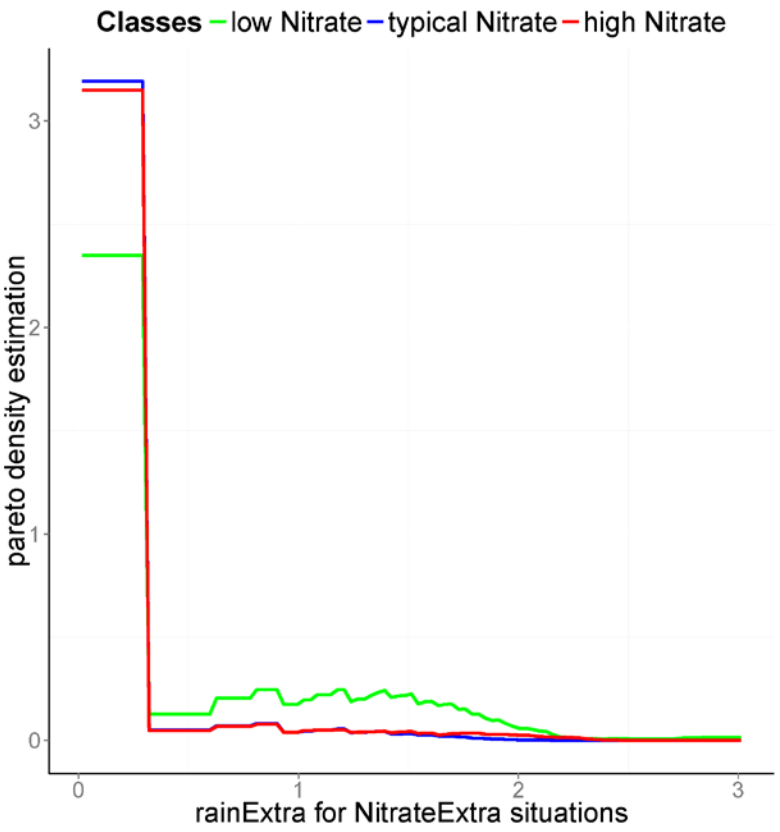

Supplement: Supplementary Information [file srep31536-s1.pdf]
